# Supplementary material for: Resolving the full spectrum of human genome variation using Linked-Reads
Source: Genome Res. 2019 Apr;29(4):635–45. doi: 10.1101/gr.234443.118 (PMC6442396; doi:10.1101/gr.234443.118)
Supplement: Supplemental Material [file supp_gr.234443.118_Supplemental_longranger-2.2.zip › longranger-2.2.tar.bz2!/lr2.2-public/lib/stan/cmdstan/stan/doc/api/html/structstd_1_1numeric__limits_3_01stan_1_1agrad_1_1var_01_4.html]

Stan: std::numeric\_limits< stan::agrad::var > Struct Template Reference


|  |  |
| --- | --- |
|  | Stan  2.5.0  probability, sampling & optimization |


- Main Page
- Related Pages
- Namespaces
- Classes
- Files

- Class List
- Class Index
- Class Hierarchy
- Class Members

All Classes Namespaces Files Functions Variables Typedefs Enumerations Enumerator Friends Macros Pages

- std
- numeric\_limits< stan::agrad::var >

Static Public Member Functions |
Static Public Attributes |
List of all members

std::numeric\_limits< stan::agrad::var > Struct Template Reference

Specialization of numeric limits for var objects.
More...

`#include <numeric_limits.hpp>`

|  |  |
| --- | --- |
| Static Public Member Functions | |
| static stan::agrad::var | min () |
|  | |
| static stan::agrad::var | max () |
|  | |
| static stan::agrad::var | epsilon () |
|  | |
| static stan::agrad::var | round\_error () |
|  | |
| static stan::agrad::var | infinity () |
|  | |
| static stan::agrad::var | quiet\_NaN () |
|  | |
| static stan::agrad::var | signaling\_NaN () |
|  | |
| static stan::agrad::var | denorm\_min () |
|  | |

|  |  |
| --- | --- |
| Static Public Attributes | |
| static const bool | is\_specialized = true |
|  | |
| static const int | digits = numeric\_limits<double>::digits |
|  | |
| static const int | digits10 = numeric\_limits<double>::digits10 |
|  | |
| static const bool | is\_signed = numeric\_limits<double>::is\_signed |
|  | |
| static const bool | is\_integer = numeric\_limits<double>::is\_integer |
|  | |
| static const bool | is\_exact = numeric\_limits<double>::is\_exact |
|  | |
| static const int | radix = numeric\_limits<double>::radix |
|  | |
| static const int | min\_exponent = numeric\_limits<double>::min\_exponent |
|  | |
| static const int | min\_exponent10 = numeric\_limits<double>::min\_exponent10 |
|  | |
| static const int | max\_exponent = numeric\_limits<double>::max\_exponent |
|  | |
| static const int | max\_exponent10 = numeric\_limits<double>::max\_exponent10 |
|  | |
| static const bool | has\_infinity = numeric\_limits<double>::has\_infinity |
|  | |
| static const bool | has\_quiet\_NaN = numeric\_limits<double>::has\_quiet\_NaN |
|  | |
| static const bool | has\_signaling\_NaN = numeric\_limits<double>::has\_signaling\_NaN |
|  | |
| static const float\_denorm\_style | has\_denorm = numeric\_limits<double>::has\_denorm |
|  | |
| static const bool | has\_denorm\_loss = numeric\_limits<double>::has\_denorm\_loss |
|  | |
| static const bool | is\_iec559 = numeric\_limits<double>::is\_iec559 |
|  | |
| static const bool | is\_bounded = numeric\_limits<double>::is\_bounded |
|  | |
| static const bool | is\_modulo = numeric\_limits<double>::is\_modulo |
|  | |
| static const bool | traps = numeric\_limits<double>::traps |
|  | |
| static const bool | tinyness\_before = numeric\_limits<double>::tinyness\_before |
|  | |
| static const float\_round\_style | round\_style = numeric\_limits<double>::round\_style |
|  | |

## Detailed Description

### template<> struct std::numeric\_limits< stan::agrad::var >

Specialization of numeric limits for var objects.

This implementation of std::numeric\_limits<stan::agrad::var> is used to treat var objects like doubles.

Definition at line 16 of file numeric\_limits.hpp.

## Member Function Documentation

|  |  |  |  |  |
| --- | --- | --- | --- | --- |
| static stan::agrad::var std::numeric\_limits< stan::agrad::var >::denorm\_min | ( |  | ) |  |

inlinestatic

Definition at line 42 of file numeric\_limits.hpp.

|  |  |  |  |  |
| --- | --- | --- | --- | --- |
| static stan::agrad::var std::numeric\_limits< stan::agrad::var >::epsilon | ( |  | ) |  |

inlinestatic

Definition at line 26 of file numeric\_limits.hpp.

|  |  |  |  |  |
| --- | --- | --- | --- | --- |
| static stan::agrad::var std::numeric\_limits< stan::agrad::var >::infinity | ( |  | ) |  |

inlinestatic

Definition at line 39 of file numeric\_limits.hpp.

|  |  |  |  |  |
| --- | --- | --- | --- | --- |
| static stan::agrad::var std::numeric\_limits< stan::agrad::var >::max | ( |  | ) |  |

inlinestatic

Definition at line 19 of file numeric\_limits.hpp.

|  |  |  |  |  |
| --- | --- | --- | --- | --- |
| static stan::agrad::var std::numeric\_limits< stan::agrad::var >::min | ( |  | ) |  |

inlinestatic

Definition at line 18 of file numeric\_limits.hpp.

|  |  |  |  |  |
| --- | --- | --- | --- | --- |
| static stan::agrad::var std::numeric\_limits< stan::agrad::var >::quiet\_NaN | ( |  | ) |  |

inlinestatic

Definition at line 40 of file numeric\_limits.hpp.

|  |  |  |  |  |
| --- | --- | --- | --- | --- |
| static stan::agrad::var std::numeric\_limits< stan::agrad::var >::round\_error | ( |  | ) |  |

inlinestatic

Definition at line 27 of file numeric\_limits.hpp.

|  |  |  |  |  |
| --- | --- | --- | --- | --- |
| static stan::agrad::var std::numeric\_limits< stan::agrad::var >::signaling\_NaN | ( |  | ) |  |

inlinestatic

Definition at line 41 of file numeric\_limits.hpp.

## Member Data Documentation

const int std::numeric\_limits< stan::agrad::var >::digits = numeric\_limits<double>::digits

static

Definition at line 20 of file numeric\_limits.hpp.

const int std::numeric\_limits< stan::agrad::var >::digits10 = numeric\_limits<double>::digits10

static

Definition at line 21 of file numeric\_limits.hpp.

const float\_denorm\_style std::numeric\_limits< stan::agrad::var >::has\_denorm = numeric\_limits<double>::has\_denorm

static

Definition at line 37 of file numeric\_limits.hpp.

const bool std::numeric\_limits< stan::agrad::var >::has\_denorm\_loss = numeric\_limits<double>::has\_denorm\_loss

static

Definition at line 38 of file numeric\_limits.hpp.

const bool std::numeric\_limits< stan::agrad::var >::has\_infinity = numeric\_limits<double>::has\_infinity

static

Definition at line 34 of file numeric\_limits.hpp.

const bool std::numeric\_limits< stan::agrad::var >::has\_quiet\_NaN = numeric\_limits<double>::has\_quiet\_NaN

static

Definition at line 35 of file numeric\_limits.hpp.

const bool std::numeric\_limits< stan::agrad::var >::has\_signaling\_NaN = numeric\_limits<double>::has\_signaling\_NaN

static

Definition at line 36 of file numeric\_limits.hpp.

const bool std::numeric\_limits< stan::agrad::var >::is\_bounded = numeric\_limits<double>::is\_bounded

static

Definition at line 45 of file numeric\_limits.hpp.

const bool std::numeric\_limits< stan::agrad::var >::is\_exact = numeric\_limits<double>::is\_exact

static

Definition at line 24 of file numeric\_limits.hpp.

const bool std::numeric\_limits< stan::agrad::var >::is\_iec559 = numeric\_limits<double>::is\_iec559

static

Definition at line 44 of file numeric\_limits.hpp.

const bool std::numeric\_limits< stan::agrad::var >::is\_integer = numeric\_limits<double>::is\_integer

static

Definition at line 23 of file numeric\_limits.hpp.

const bool std::numeric\_limits< stan::agrad::var >::is\_modulo = numeric\_limits<double>::is\_modulo

static

Definition at line 46 of file numeric\_limits.hpp.

const bool std::numeric\_limits< stan::agrad::var >::is\_signed = numeric\_limits<double>::is\_signed

static

Definition at line 22 of file numeric\_limits.hpp.

const bool std::numeric\_limits< stan::agrad::var >::is\_specialized = true

static

Definition at line 17 of file numeric\_limits.hpp.

const int std::numeric\_limits< stan::agrad::var >::max\_exponent = numeric\_limits<double>::max\_exponent

static

Definition at line 31 of file numeric\_limits.hpp.

const int std::numeric\_limits< stan::agrad::var >::max\_exponent10 = numeric\_limits<double>::max\_exponent10

static

Definition at line 32 of file numeric\_limits.hpp.

const int std::numeric\_limits< stan::agrad::var >::min\_exponent = numeric\_limits<double>::min\_exponent

static

Definition at line 29 of file numeric\_limits.hpp.

const int std::numeric\_limits< stan::agrad::var >::min\_exponent10 = numeric\_limits<double>::min\_exponent10

static

Definition at line 30 of file numeric\_limits.hpp.

const int std::numeric\_limits< stan::agrad::var >::radix = numeric\_limits<double>::radix

static

Definition at line 25 of file numeric\_limits.hpp.

const float\_round\_style std::numeric\_limits< stan::agrad::var >::round\_style = numeric\_limits<double>::round\_style

static

Definition at line 50 of file numeric\_limits.hpp.

const bool std::numeric\_limits< stan::agrad::var >::tinyness\_before = numeric\_limits<double>::tinyness\_before

static

Definition at line 49 of file numeric\_limits.hpp.

const bool std::numeric\_limits< stan::agrad::var >::traps = numeric\_limits<double>::traps

static

Definition at line 48 of file numeric\_limits.hpp.

---

The documentation for this struct was generated from the following file:

- src/stan/agrad/rev/numeric\_limits.hpp


---

[ Stan Home Page ]

*© 2011–2014,
Stan Development Team.*
